# Myths, misconceptions, othering and stigmatizing responses to Covid-19 in South Africa: A rapid qualitative assessment

**DOI:** 10.1371/journal.pone.0244420

**Published:** 2020-12-22

**Authors:** Tenielle Schmidt, Allanise Cloete, Adlai Davids, Lehlogonolo Makola, Nokubonga Zondi, Monalisa Jantjies

**Affiliations:** 1 Human and Social Capabilities Division, the Human Sciences Research Council (HSRC), Pretoria, South Africa; 2 Faculty of Health Sciences, Nelson Mandela University, Port Elizabeth, South Africa; 3 Children’s Institute of the University of Cape Town (UCT), Cape Town, South Africa; University of Toronto, CANADA

## Abstract

Severe Acute Respiratory Syndrome Coronavirus 2 (SARS-CoV-2) is a new strain of virus in the Coronavirus family that has not been previously identified. Since SARS-CoV-2 is a new virus, everyone is at risk of catching the Coronavirus disease 2019 (Covid-19). No one has immunity to the virus. Despite this, misconceptions about specific groups of people who are immune to Covid-19 emerged with the onset of the pandemic. This paper explores South African communities’ misconceptions about who is most vulnerable to Covid-19. A rapid qualitative assessment was conducted remotely in Gauteng, KwaZulu-Natal and the Western Cape provinces of South Africa. Recruitment of study participants took place through established relationships with civil society organizations and contacts made by researchers. In total, 60 key informant interviews and one focus group discussion was conducted. Atlas.ti.8 Windows was used to facilitate qualitative data analysis. The qualitative data was coded, and thematic analysis used to identify themes. The results show a high level of awareness and knowledge of the transmission and prevention of SARS-CoV-2. Qualitative data revealed that there is awareness of elderly people and those with immunocompromised conditions being more vulnerable to catching Covid-19. However, misconceptions of being protected against the virus or having low or no risk were also evident in the data. We found that false information circulated on social media not only instigated confusion, fear and panic, but also contributed to the construction of misconceptions, othering and stigmatizing responses to Covid-19. The study findings bring attention to the importance of developing communication materials adapted to specific communities to help reduce misconceptions, othering and stigmatization around Covid-19.

## Introduction

In December 2019, Severe Acute Respiratory Syndrome Coronavirus 2 (*SARS-CoV-2*), the strain of Coronavirus that causes Coronavirus disease 2019 (Covid-19) was identified in Wuhan, China [1–5]. The first imported and locally transmitted cases of Covid-19 in South Africa were detected in March 2020 [6]. Most cases at that time were people returning from overseas travel mainly from countries in Europe. On 15 March 2020, the South African government declared a national state of disaster [7].

To prevent community transmission of the disease the South African government announced stringent restrictions based on an initial 21-day stay-at-home national lockdown starting from 26 March 2020 that extended indefinitely even though some restrictions were relaxed based on risk mitigation considerations [8]. During the 21-day stay-at-home national lockdown, movement was restricted to essential service personnel such as medical doctors, nurses, police and grocery store staff [8]. Those requiring medical care, or shopping for essentials were allowed to leave their homes for restricted periods or under strictly controlled conditions [6, 8, 9]. In addition to this, the government deployed the South African National Defence Force (SANDF) to support the South African Police Services (SAPS) in managing compliance to the lockdown regulations. These approaches helped with the containment of Covid-19 and allowed greater responsiveness and preparedness of already overstretched health systems [10, 11]. However, in the first nine days of October 2020, South Africa was ranked 10^th^ in the world [12], recording 686 891 people who have Covid-19, 17 408 deaths and 618 771 recoveries [6].

With such an accelerated increase in Covid-19 cases and with the absence of a vaccine, widespread myths and misconceptions with regards to the transmission of Covid-19 also sparked an ‘infodemic’ according to the World Health Organization (WHO) [13]. Myths and misconceptions emerge with disease outbreaks and Covid-19 proved to be no different [14–16]. In our study we use the terms myths and misconceptions interchangeably, given that these concepts are so used in the literature [17]. Myths and misconceptions may refer to ideas and concepts believed or held by a group of people, which are not scientifically validated [18, 19]. Identifying myths and misconceptions is crucial in disease outbreak, since these might affect preventive and containment measures. To our knowledge, research on myths and misconceptions about Covid-19 in South Africa is undocumented.

Researchers investigating Covid-19 knowledge, attitudes, and practices (KAPs) of residents in Hubei, China found that most of the study sample had correct knowledge about Covid-19 (90%) and maintain appropriate protective practices towards Covid-19 [20]. Singh and colleagues (2020), tracked social media and identified myths ranging from Covid-19 home remedies to conspiracy theories about the origin of the virus, to misinformation that warm weather kills the virus [21]. In a cross sectional survey, conducted in the United States of America, results showed that participants who identified as ‘black’, had low health literacy, were more likely to be less worried about Covid-19, believed that they would not become infected and felt less prepared for an outbreak than those who are more vulnerable to complications of infection because of age and comorbid conditions [22]. According to Zhong and colleagues (2020), people’s adherence to preventive measures is affected by their KAPs towards Covid-19 [20]. Thus correct knowledge and awareness of protective measures impact on adherence to Covid-19 specific containment measures.

Similarly, the fear of SARS-Cov-2 infection also increases social exclusion and stigmatization [23]. Stigma is a well-documented barrier to health seeking behavior, engagement in care and adherence to treatment across a range of health conditions globally [24]. A small number of studies conducted on Covid-19 stigma in China and some European countries revealed that the disease has led to the stigmatization and discrimination towards various groups of people [25]. The groups most affected by stigma include healthcare workers caring for those affected by Covid-19 [26], people who have recovered from Covid-19 [27, 28], those belonging to lower socioeconomic groups and those having particular religious and racial identities [27, 29]. In correspondence in the *Lancet*, Devakumar and colleagues (2020) reported that with the transmission of Covid-19 from China, discrimination towards Chinese people increased [30]. According to Bagcchi (2020) stigma associated with Covid-19 poses a serious threat to the lives of healthcare workers, people who are recovering from Covid-19, and people who have Covid-19 [31].

Lessons learnt from other pandemics such as HIV might prove to be helpful to understand how stigma facilitates the transmission of Covid-19. SARS-CoV-2 and HIV are by virtue of its transmission vastly different [32]. However, South Africa’s initial response to HIV bears crucial lessons for the management of the Covid-19 pandemic in South Africa. Globally, HIV-related stigma, has been cited as one of the most enduring barriers to ending the HIV pandemic [33, 34]. With the onset of the HIV pandemic, a discourse of othering that mediates cultural and racial positionings concerning those deemed responsible for transmitting HIV and thus were considered more vulnerable to HIV infection emerged in South Africa [33]. The “other” in South Africa is defined as someone with a religion or ethnic group different to one’s own, and gay men who are blamed for the transmission of HIV [35–38]. HIV was first diagnosed among ‘white’ gay-identified men who have sex with men (MSM) in the 1980s [39], and only after 1990 did a second heterosexually-transmitted epidemic emerge among South Africa's ‘black’ population [40]. With the first confirmed cases amongst MSM, HIV was perceived as a “gay-disease” [40, 41].

The history of stigma and discrimination in South Africa is of course most evident with the *apartheid* system of legislated segregation [42]. The *apartheid* regime created separation between different racial categories and informed a discourse of shame that was internalised by those classified by the *apartheid* system as ‘black’ and ‘coloured’ [43, 44]. Even though the South African *apartheid* regime assigned superiority to ‘whiteness’ and an inferior status to notions of ‘blackness’, was abolished, the social constructions of race imposed onto South Africans is still used to organise our everyday lives [45]. Thus, as Campbell and colleagues (2005) in their seminal research on HIV-related stigma remind us that “at the symbolic level, the close link that several informants made [in their study] between HIV and ‘black’ African people both draw on and feeds back into negative stereotypes of ‘black’ people that have a long history in South Africa” (p.813) [46]. In our paper, we document how narratives of race (and class) premised on *apartheid* ideologies inform Covid-19 stigmatizing responses.

## Study aim

The aim of this study is to investigate South African communities’ constructions of myths and misconceptions about who is most vulnerable to Covid-19 and how at times these beliefs inform a discourse of stigmatization and othering.

## Materials and methods

### Study design

The overall aim of this study was met using an exploratory descriptive qualitative design. An exploratory descriptive qualitative design is particularly relevant where information is required directly from those experiencing the phenomenon under investigation and where time and resources are limited [47, 48]. An exploratory descriptive qualitative design provides an insider perspective to the phenomenon under study.

### Ethical considerations

Ethics approval was obtained from South Africa’s Human Sciences Research Council (HSRC), Research Ethics Committee (REC) (Protocol No REC 5/03/20).

### Study participants and setting

Study participants were identified from various socio-cultural and economic backgrounds within Gauteng, KwaZulu-Natal and the Western Cape provinces of South Africa. These provinces had the highest burden of Covid-19 at the time of the rapid assessment. Eligible participants were 18 years of age or older.

In total, 60 key informant interviews and one focus group discussion was conducted. With regards to the gender breakdown: 36 cisgender women, 22 cisgender men and two transgender women took part in key informant interviews. The focus group discussion comprised of two cisgender women and three cisgender men.

Interviews were conducted with the following key informants: Minibus—taxi owners/drivers/commuters (n = 6); community health workers (n = 6); faith-based leaders (n = 3); traditional leaders (n = 2); educators (n = 3); sex workers (n = 2); people living with HIV (n = 3); person living with diabetes mellitus (n = 1); sexual and gender minority group members (n = 4); relief aid workers providing services to homeless people (n = 2); relief aid workers providing services to migrant communities, a leader of a migrant umbrella NGO and a migrant domestic worker (n = 5); people with disabilities (n = 6); *shebeen* (an informal drinking place) patrons (n = 5); out of school youth (n = 6); airport workers (n = 3) and a pregnant woman (n = 1). Interviews were also conducted with old age home carers, resident in Gauteng (n = 1) and in the Western Cape (n = 1). One focus group discussion was conducted remotely with five staff members managing an old age home in KwaZulu-Natal.

### Sampling procedures

**Fig 1:**
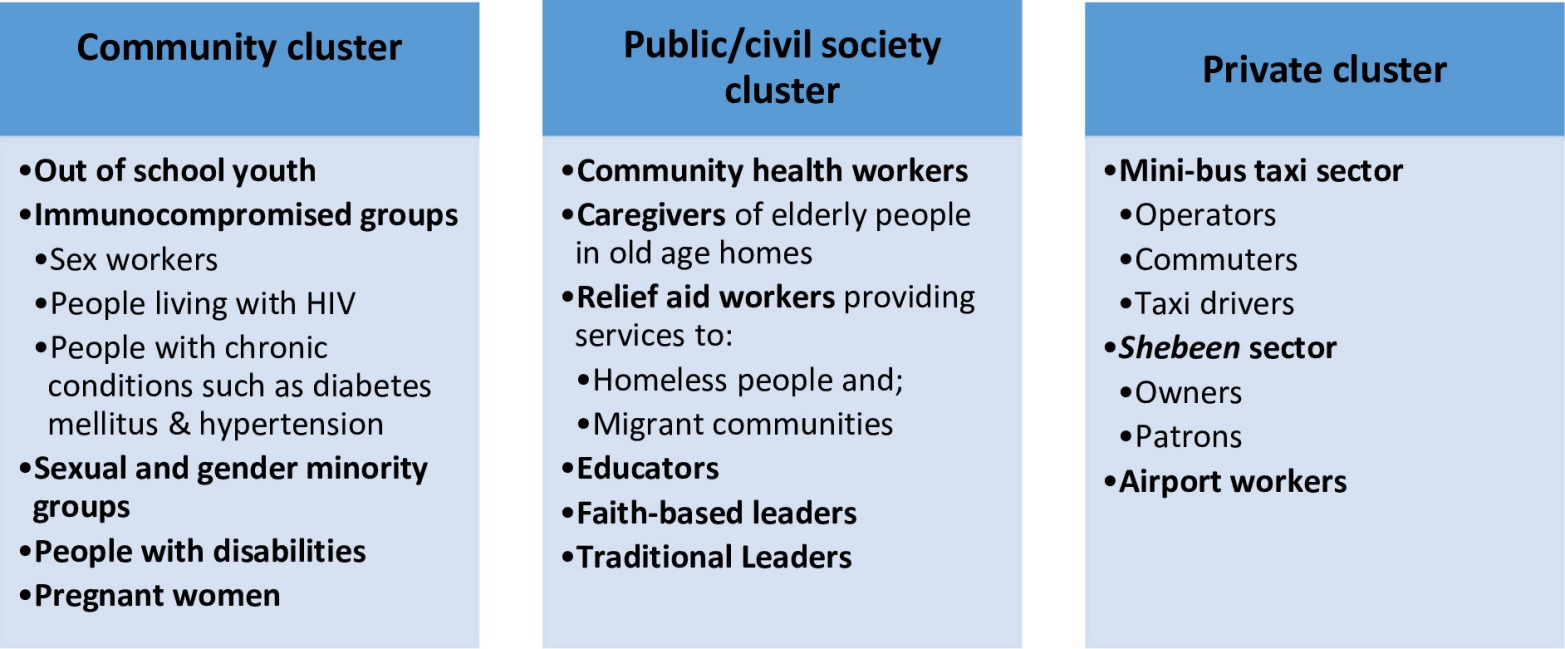
Target population.

Purposive sampling was used to identify and select participants to take part in the study. In order to ensure diversity, we purposively selected key informants representing various sectors of South African society. The target population was categorized into the following groups: namely the community cluster, public/civil society and private sector cluster. (see Fig 1)

Research staff identified key informants from the three high-burden provinces through pre-existing relationships with non-governmental organizations (NGOs) and civil society organizations that provide services to vulnerable groups. For example, the elderly (aged 70 years and older) and persons (any age group) who have serious underlying health conditions (e.g. hypertension, diabetes, cardiovascular disease, chronic respiratory disease and cancer) are considered to be higher risk for severe illness from Covid-19 [49–51] were purposively selected. Other immunocompromised groups included people living with HIV (PLHIV), sex workers, and people with disabilities. Other socially marginalized groups such as undocumented workers and migrant workers were also selected to take part in the study. It was important to include both members of the general public and representatives of specific populations in order to obtain diversity of experiences of the Covid-19 pandemic.

### Data collection and tools

Telephonic interviews were used to collect data. Data collection was conducted from 5 to 18 April 2020. With the onset of the Covid-19 pandemic, as per regulations set by the REC of the HSRC, all non-interventional research with human participants were suspended or switched from face-to-face to remote (i.e. online, telephonic) data collection. The ethically approved study information sheet and consent forms were either emailed or sent to participants via WhatsApp. Before the start of the interview, informed consent was obtained verbally from all participants. After having read and understood the contents of the study, the participants chose their own pseudonym. Verbal consent to participate in the study was audio-recorded as part of the interview process. On average, telephonic interviews lasted approximately 30–45 minutes depending on the length of responses.

Semi-structured interview guides were used to conduct interviews (see S1 File). The following themes were explored in key informant interviews and in the focus group discussion: (i) Basic knowledge of the transmission of Covid-19; (ii) Sources of information about Covid-19; (iii) Myths, misconceptions, false information about the transmission of the disease (iv) Notions of vulnerability (vii) Perceptions/experiences of the lockdown and social distancing. The development of the themes explored in key informant interviews and in the focus group discussion was in the first instance, informed by the overall aim of the study as well as the study objectives which included amongst others, the following: i) To gather knowledge, attitudes, perceptions, and behaviours of the general public and specific populations about Covid-19; ii) To understand public perceptions and constructions of Covid-19 risk; iii) To explore the level of stigmatization and ‘othering’ as it relates to the Covid-19 pandemic.

Interviews were conducted and audio-recorded in English, isiXhosa, isiZulu and Afrikaans as these are predominant languages spoken in each of the three provinces. Each researcher made use of their personal cellular telephones to audio record interviews. Once an interview was arranged, the researcher called the participant and switch their cellular telephone to speaker mode. The researcher then informed the participant of the recording clause in the informed consent form. Once the participant agreed to have the interview recorded, the researcher switched on the recording application on their laptops and place their cellular phone at an appropriate distance to the laptop. The researcher, at this point, obtain verbal consent for the interview. Once the interview was completed, the researcher would stop the recording, and the file renamed using a predetermined file name to ensure confidentiality. A standard operating procedure manual outlining these procedures as well as online training were provided to all researchers.

Fourteen researchers employed at the HSRC, all trained in qualitative data collection methodologies conducted the interviews. Researchers were proficient in each of the major languages spoken in the provinces where the study took place. In each of the participating provinces, nine cisgender women and five cisgender men conducted key informant interviews, with one of the 14 researchers facilitating the focus group discussion. Of the 14 researchers, four completed doctorates in public health, anthropology, sociology and research psychology. The remaining 10 were all Masters-level educated researchers.

### Data analysis

All interviews were transcribed verbatim and translated into English, by investigators who were part of the study. Data was de-identified during the translation and transcription process. Thereafter members of the core research team verified that indeed all-identifying information were removed from all transcripts. We used ATLASti.8 Windows to facilitate qualitative data analysis.

AC together with one of the principal investigators of the study led the data analysis process. AC assigned preliminary codes, and then further categorization and sub-grouping was undertaken by TS and AD. AC developed an initial thematic, content-based codebook using ATLASti.8 Windows. The following steps were followed during the qualitative data analysis process: Firstly, each transcript was read and re-read. Open coding was used to identify themes. This process enables the researcher to use the participants own words and phrases, without the interference of preconceived classifications. Similar codes were grouped together and categorized, the categories and codes were then compared in order to generate an analytical schema and to facilitate the interpretation of the findings.

## Results

### Knowledge of Covid-19 and awareness of who is most vulnerable

Study participants generally understood that Covid-19 is a respiratory disease, as is clear in the excerpt below from an interview conducted with a traditional leader.

“I know that Corona is a flu-like virus. It is kind of a fever that attacks your lungs. If your immune system is compromised, you are at risk of contracting it. It is transferred through droplets and if maybe you touch a place, where somebody that is infected by it may have coughed and there were droplets that were there. If you touched there and then touch your face, any opening on your face, there is a risk of contracting it. I think for you to be safe you [must] wash your hands, cover your nose and keep a distance from other people. I think in a nutshell that is what it is” (Male, traditional leader: Gauteng)

The participant also demonstrated to have knowledge of best practices to prevent infection and transmission of Covid-19. Overall, study participants demonstrated correct knowledge and awareness of Covid-19. This includes knowledge on how the virus is transmitted and how infection can be prevented through social distancing and the use of personal protective equipment (PPE) such as gloves and masks coupled with strict hand washing (see Table 1).

**Table 1:** Knowledge of Covid-19.

Definition of theme	Illustrative quotations
Correct knowledge and awareness of Covid-19	“What I have heard about the coronavirus is that it is a flu type virus which affects the respiratory system. It started out in China, somewhere in Wuhan and if you come into contact with somebody else you could be infected by the virus, by touching them or through mucus” (Transgender woman, sexual and gender minority group member: Gauteng)
	“”…[B]ecause of the nature of this virus, we were alerted by it being very highly contagious. It manifested itself with similar symptoms to flu, namely the high temperatures, the difficulty in breathing and the coughing. Along the way we discovered that it is spread by certain habits or human habits are causing it and so on. Then we began to take note. I also discovered that it is only when you get in close proximity to somebody and not take the necessary precautions that this virus can spread very rapidly” (Female, person living with diabetes mellitus: KwaZulu-Natal)
	“I know that corona is there and [it] is a pandemic. It spreads fast for example a person sneeze on the hand and touches someone without washing their hands first. And it enters the body by eyes, nose and mouth. It goes to the throat from there to the oesophagus and it goes to the lung that is when the person gets difficulties in breathing” (Female, communtiy health worker: Western Cape)
	“It’s a new disease I heard about it. It is a disease where you have it you are not able to breath nicely, you cough, you get the headaches, you get the fever. If you get those signs, you must phone the doctor and go to the nearest clinic if you are able to do so” (Female, relief aid worker providing services to migrant communities: KwaZulu-Natal)

Similarly, study participants’ awareness of risks was also highlighted in terms of the following: Physical contact with those who are infected, be it outside their homes, in public transport, or by touching contaminated surfaces like the money that is passed around by passengers in a minibus-taxi. Awareness of preventive measures are demonstrated in the following quotations:

“I've heard about it yes, that it's contagious, you have to cover your mouth, there's no handshake involved. If you cough, you use your elbow and you must always use sanitizer after everything that you do. Keep your surfaces clean like the door handle, [keep] everything clean, keep on bleaching or [using] *Jik* [i.e. bleach] or anything else. The lockdown you [must] stay indoors. I think it’s for 21 days in order to avoid getting in touch with people, in case somebody has it on the outside so if you’re indoors you will be safe from getting the virus” (Female, sex worker: Gauteng)

“Yes I use public transport…with public transport you have no choice, I mean you are in one [minibus] taxi obviously you have to pass someone the money or someone has to pass the money to you. I just try not to touch my face and nose, as soon as I get where I am going, I make sure I wash my hands, because I do not have gloves. I use a mask because we got masks at work. In a [minibus] taxi; I also have the sanitizer for my hands as well*”* (Female, person with disabilities: Western Cape)

Study participants were aware that older people and those with immunocompromised conditions are more vulnerable to catching the virus (see Table 2):

“It would be my mother-in-law because she is in her 80s. She is in an old-age unit, they are on lockdown. They can't even come up and have their breakfast and lunch. So she's the one who I am mostly concerned about” (Female, person with disabilities: Gauteng)

“I am taking it seriously because I live with my gran and she’s a 67-year-old lady, so they are more vulnerable to this disease” (Female, out of school youth: KwaZulu-Natal)

“The way that I understand it [for] people who are living with HIV and TB, that it's very dangerous for them, so people [who] live with these illnesses that it can also cause your death and so [I am concerned about] myself who is HIV-positive” (Female, person living with HIV: Western Cape)

**Table 2:** Awareness of who is most vulnerable to Covid-19.

Definition of theme	Illustrative quotations
Awareness of who is most vulnerable to Covid-19	“Well, especially the seniors in my community and obviously those who are vulnerable. You know those who are terminally ill and who have people in their places of dwelling or in their homes, you know that have to go out and then come back and infect them, as it were so that is a concern” (Male, faith-based leader: Western Cape)
	“The risk is higher with the elderly around the ages of 60 and 65 because they have underlying conditions such as diabetes, asthma and their immune systems are compromised. So it is important to ensure that people are safe as it will be difficult for the elderly to access clinics. Some of them have to get medication so we suggest that health[care] workers bring medications to the patients place of residence so that people can stay at home” (Male, traditional leader: KwaZulu-Natal)
	“My brother is over 60, he does have [a] heart problem, so my concern is [with] him because they are more [at risk] as their immune system is very weak. So the worry was [with] him and my sister, so [I] call them all the time [and] ask if they are eating healthy. And [if] they do have slight cough and fever they must see specialist. So my big concern was them. And basically the communities also, they are also part of our families, I always keep checking if our old people are fine. Even my neighbor I greet and ask, are you okay? Because [as] healthcare workers, that is what we do, we check on our community” (Female, community health worker: KwaZulu-Natal)

Even though study participants reported that the elderly and those with immunocompromised conditions are more vulnerable, a level of awareness that everyone is at risk of being infected was also evident in the data.

### Covid-19 false information, myths and misconceptions

In the weeks following the announcement of the lockdown, the South African government announced the implementation of a mass door-to-door screening and testing campaign. At the same time, false information was also distributed on social media warning South African communities to refrain from testing (see Table 3).

“Now, they have people around, testing. The internet says; don't allow the testing and then the other people said they should allow the testing. So that for me is [the] very confusing part. So, I don't know what to do if people are doing door-to-door testing. You do not know whether to welcome them or to check them or chase them away. Then some people say don't open the door [for] them, they will give you the Coronavirus and so that's where you're confused. What should we do because we don't know, and the internet is not always true?” (Female, sex worker: Gauteng)

“…The other [thing that they are] say[ing] [is], don’t accept any test for Corona because it’s already infected” (Female, relief aid worker providing services to migrant communities: KwaZulu-Natal)

**Table 3:** False information.

Definition of theme	Illustrative quotations
False information about Covid-19	“They talk about it they are so afraid they say maybe I am going to die. We don't know maybe God is angry people just say a lot of things some they say coronavirus is affected through washing in the same dish. But when I later heard in the TV it's not transferred by washing in the same bathroom it is spreading through coughing or sneezing yes” (Female, sex worker: Gauteng)
	“Really there's a lot I heard the other day I was sitting in the house. The one neighbour said did you hear [about] the Advil pain tablets, to me it was just so simple [laughs].Because the neighbour was serious and I use Advil now and then how, can Corona be in this tablet?” (Female, person living with HIV: Western Cape)
	“Yes, I think you saw the guy arrested yesterday, a video circulating on Whatsapp a man saying they test with contaminated testing kits. People must not test, now we don’t know what is the truth” (Male, person with disabilities, Western Cape)

False information distributed on social media also caused confusion, fear and panic amongst South African communities. In some instances, healthcare workers reported that the distribution of false information influenced public responses to the screening and testing campaign:

“Like as clinic staff we go in door- to-door, there are incidences where a house owner would refuse for us to go in, saying we don’t want your vaccines because they have Corona. Then we had to explain that we are not injecting people, we are just screening and asking questions. People are really scared, because of what they heard…” (Female, community health worker: Western Cape)

Moreover, false information about the availability of a Covid-19 vaccine also circulated on social media. Study participants mentioned that in the communities in which they live, rumors spread of a vaccine that was released for Covid-19:

“Another myth is that there is a vaccine. I do not know whether that is true, but apparently there is a vaccine and people need to be vaccinated for Coronavirus” (Female, old age home carer: Gauteng)

In addition to false information about the availablity of a Covid-19 vaccine, home remedies, such as drinking hot water, was seen as a cure for the disease:

“We drink hot water. Some videos said that we must try and drink hot things. If you come into contact with the virus and [it’s] still in your throat you can drink water. It is possible that it can be washed into the stomach and the stomach will kill the virus” (Male, community health worker: KwaZulu-Natal)

Parallel to false information of the availablity of a Covid-19 vaccine, were findings suggesting that eating the right things, in this instance maize (corn) meal porridge might serve as a protective factor to Covid-19:

“I also believe that people in our country, or in Africa doesn’t see the severity of it, they believe that they are immune from it. Just as an example, I was in a Uber, before the lockdown started and I made a conversation about [Covid-19] and I asked the driver is he worried about getting infected. And so his response to me was Oh no I won’t get the virus because I eat *pap* [translated from Afrikaans, (i.e. local language) to English, means porridge, referring to maize meal traditionally made]. And I ask him what do you mean and he says, because my system is strong it will only get people who don’t eat *pap*, who are weak.So eating *pap*, the whole perception of being healthy is misconstrued there in terms of *pap* for some people *pap* is a healthy lifestyle” (Transgender woman: Sexual and gender minority group member, Gauteng)

Inasmuch as qualitative findings point to study participants having correct knowledge and awareness regarding the transmission and prevention of Covid-19, myths and misconceptions about the disease was also evident in the data (see Table 4).

**Table 4:** Myths and misconceptions about who is most vulnerable to Covid-19.

Definition of theme	Illustrative quotations
Myths	“I know that it cannot survive in [a] hot environment it requires a cold environment and it also spread[s] through saliva” (Male, taxi driver: Gauteng)
Misconceptions about who is most vulnerable to Covid-19	“People first thought that it was the virus of the sick. Especially those people who are living in the rural areas and the notion that the virus does not necessarily attack people that are ‘black’ in the name, so that was the attitude of many people, but also lack of information and arrogance of our people” (Male, traditional leader: Gauteng)
	“Just thought that if I'm not an old person if I haven't travelled overseas that means that I wouldn't get it that was the case” (Pregnant Woman: Gauteng)
	“So, for me that’s fake but it’s been going around in videos and voice notes. There was also something that I heard [that] it’s not attacking, ‘black’ people, ‘black’ raced people and stuff like that, that’s what I’ve been hearing” (Female, out of school youth: KwaZulu-Natal)
	“There [are] people saying oh no, this sickness don’t affect ‘black’ people!” (Female, relief aid worker for migrant communties, KwaZulu-Natal)
	“I’ve got some friends in Eastern Cape, who don’t [abide] by the rules and they always making fun about we won’t get coronavirus it is for ‘whites’ only so they think it is a joke” (Male, person with disabilities: Western Cape)

In the first instance, study participants revealed that young people do not see themselves at risk of becoming infected with the virus that causes Covid-19. Hence, they raised concerns that the youth were not taking the Covid-19 pandemic seriously:

“My issue is that the younger ones are not taking it seriously. Even though it will mostly kill the elderly since their immune system is compromised, the young and middle aged are not taking it seriously. They think it is one of those things that will just pass but the most vulnerable and most at risk are the elderly and the young ones because their immune system is still weak, it is compromised. Those are the people who are often sick. But it is mostly going to affect and kill elderly people” (Male, faith based leader: Gauteng)

Myths that the virus is man-made, originating from “eating the wrong things” and transmitted via 5G technology were also observed in the data:

“I’ve heard quite a lot, very confusing, some legit, some not legit. Basically it’s a virus that attacks your lungs and flu-like symptoms. Some people think that its man-made, some people think that it’s due to people eating the wrong things!” (Female, out of school youth: KwaZulu-Natal)

“Ok let me start about the 5G…I keep asking myself, how can I get infected with 5G…how do I get infected by it. Because I believe I only get it by touching someone who has the virus and then maybe I will touch my face you see. So this thing of 5G…” (Female, person living with disability: Western Cape)

Myths and misconceptions regarding the zoonotic origin of Covid-19 also emerged from the data:

“I even heard that the virus is associated with bats and snakes” (Male, community health worker: KwaZulu-Natal)

“Ay, *ja* I saw in the media, social media, it was yesterday I think [it] was a ‘white’ man, I think it was in the hospital, there was something like a worm from [his] lip it was a doctor taking out the worm from the lip they called it, it’s a Coronavirus, I will forward it to you…”” (Female, person with a disability: KwaZulu-Natal)

At the other end of the spectrum, data revealed that there is a discourse of Covid-19 denialism:

“…“…Firstly it will go back to the issue of misconception. You will have people who will come up with their own theories and about [the] virus, you will even get people who says there is no virus” (Female, *shebeen* patron: Western Cape)

Myths and misconceptions about who is more vulnerable to Covid-19 were based on who was first diagnosed with SARS-CoV-2 in South Africa. Travelers from Italy were the first group to be diagnosed with SARS-CoV-2 in South Africa.

Similarly, those considered to be wealthy were more vulnerable to becoming infected with Covid-19, than those from poorer communities:

“It [Covid-19] does not affect the poor. It affects the rich because obviously in South Africa it came through a person coming from overseas” (Female, person living with HIV: KwaZulu-Natal)

“Those from the township … okay, think the virus is for the middle class, upper class and that they cannot get the virus because they do not travel to other countries and are not tourists. They also believe that this virus is for wealthy people, who have money and ‘white’ people, not Africans or ‘blacks’” (Female, person with disabilities: Western Cape)

Misconceptions that people of a higher socio-economic status were perceived to be more vulnerable to SARS-CoV-2 than others appeared to be common across key informant interviews. Having travelled overseas, race and class were constructed as protective factors against Covid-19. Claims that the disease is a “white-man’s disease”, were influenced by assertions that those assigned ‘black’ in South Africa are naturally immune to the disease.

“There is a perception that again it is a ‘white’ person disease because ‘white’ people, and I say that in inverted commas are, the ones who travel overseas and they brought it to Africa. Again my perception is there is a lot of knowledge and understanding that it’s not about ‘white’ people who also travel ‘black’ people also travel, when we first found out about the first lot of patients in KwaZulu-Natal. I know that one of the people who has gone overseas was an African woman in my mind are these people not educating themselves enough or do they just block out of their mind that this was an African woman who went there. There perhaps it becomes a class thing, lower class people don’t look at it as if they would [because it is] upper class people who travel a lot” (Transgender woman, sexual and gender minority group member: Gauteng)

In the quotations that follow, study participants refer to notions of natural immunity from Covid-19:

“At first, I'm [going to] sound racist but they claim that only people who are ‘white’ will be infected by people who are mostly interacting with [‘white’ people]. Plus, that only ‘white’ people can get infected” (Pregnant woman: Gauteng)

“Some people used to say that it can’t affect ‘black’ people. They thought it was for ‘white’ people. Ja that was mostly [what was said] out here that it doesn’t affect ‘black’ people, we have strong genes and so on” (Male, community health worker: KwaZulu-Natal)

“People are taking it as a joke, it’s lockdown but people are still all over. They only think it will affect the old people with chronic diseases alone, not the young, youth. They don’t want to wash [their] hands, [they] don’t want to cover when one is coughing or sneezing, it’s supposed to be a lifestyle thing not to do it because there is Corona. They also believe it was not meant for Africans, it was for China and Italy and all those stories*”* (Female, community health worker: Western Cape)

Such misconceptions informed othering and stigmatizing responses to Covid-19.

### Othering and stigmatizing responses to Covid-19

Misconceptions about natural immunity to the virus further informs a discourse of othering and stigmatization. Qualitative data revealed that people of Asian descent were blamed for the transmission of the disease:

“Also a lot of people said it was from the Chinese people, the virus comes from them, [be]cause of what they eat and all of that” (Transgender woman, sexual and gender minority group member: Gauteng)

“It’s going to be stigma towards them. Like the time of HIV. People who are [living with] HIV are still stigmatized in our society. So this is the same thing with the Coronavirus. In Gugulethu (name of a township in Cape Town) mall, after people found out that the virus was from China [they stopped] going to [the] China store” (Female, *shebeen* patron: Western Cape)

In addition to the stigmatizing responses towards people of Asian decent with the onset of the Covid-19 pandemic, study participants also made an association between HIV-related stigma and the stigma attached to Covid-19:

“There is a stigma attached to it. Let's keep it to ourselves. No one should know that, you know. Yeah, I think it is the same thing. We reacted [the same way] when people were getting AIDS in our communities” (Male, airport worker: KwaZulu-Natal)

“I think obviously discrimination and stigma will be the main thing. Because I will make an example of HIV in the block of the flats that I live in. People are still…very discriminatory against people who are living with HIV. So with Coronavirus you know I am sure that person will be excluded from everything…” (Female, person living with HIV, KwaZulu-Natal)

## Discussion

In our study, participants draw parallels between HIV-related stigma and stigmatizing responses to Covid-19 as the disease unfolded in South Africa. Although great strides have been made in the management of the HIV pandemic in South Africa, HIV-related stigma continue to be serious barriers to HIV testing, prevention, access to treatment and care for PLHIV, and the mitigation of impact of the disease. Importantly, South Africa is home to the largest population of PLHIV [52]. Historically, the AIDS denialism discourse (of beetroot, garlic and lemon) [53] that characterised the HIV pandemic in South Africa was further complicated by an othering of blame for the transmission of the disease premised on racism, patriarchy and homophobia [33].

Moreover, the South African government’s claim then, in contrast to scientific consensus, that HIV was not the cause of AIDS and that antiretroviral (ARV) drugs were not useful for patients, lead to a refusal of donated nevirapine medication, as well as grants from the Global Fund [54]. As a consequence, according to Chigwedere and colleagues (2008), more than 330 000 lives were lost because a feasible and timely ARV treatment program was not implemented in South Africa [54].

Prof Abdool Karim, one of the scientists vocally opposed to the South African government’s response to the AIDS pandemic, is now co-chairing the National Coronavirus Command Council [55]. With less than 1000 people who have Covid-19, the South African government responded swiftly and implemented one of the world’s strictest lockdowns [56]. This early intervention and swift response delayed the Covid-19 peak, according to Prof Abdool Karim [55]. Thus, the most obvious lesson between how the two pandemics were managed is the difference an early response can make [57]. Crucial to South Africa’s response to the Covid-19 pandemic is putting science above politics [57]. In this way, according to Mark Heywood (2020), a South African AIDS activist, the government’s prompt and scientific response to Covid-19 means that the battle is not as intense as when HIV was “turned into a political fight” (p.1) [57]. With HIV, according to Mia Malan, editor-in-chief of the Bhekisisa Centre for Health Journalism, “the government started an evidence-based response much too late.” (p.1) [57].

HIV-related stigma remains complicated because of the persistence of myths and misconceptions. According to Mwamwenda (2010), myths and misconceptions that continue to be persistent with HIV, include amongst others the following: “HIV is a disease of ‘black’ people; HIV was the creation of people who wanted to exterminate ‘black’ people, such as Africans, African Americans and homosexuals; God sent HIV as a means of curbing or destroying sexual immorality and HIV is transmitted through mosquitoes” (p.118) [58].

In our study, not only were wealthy ‘white’ South Africans blamed for the transmission of the disease but also people from Asian descent. With the onset of the Covid-19 pandemic in South Africa, overseas travellers were among the first people who acquired Covid-19. As a result, overseas travellers were blamed for the transmission of the disease and were considered more vulnerable to infection. In this way, Covid-19 was considered a ‘foreign import’ [59]. Mediated by racial positionings, an association between those who can afford overseas travel, meant that ‘white’, wealthy South Africans were not only to blame for the transmission of the disease, but were also considered to be more vulnerable to infection. Our study findings revealed that Covid-19 was perceived as a “white-man’s disease”. Consequently, those categorised as ‘black’ perceived themselves not to be at risk of catching Covid-19.

Similar findings were reported in a study conducted in Nigeria; views included that the virus is a “rich man’s disease” that cannot affect the poor [60]. Compared to other continents, in Africa, some of the verbal and physical acts of stigmatization are unique only in that they are targeted at both groups that are usually seen as privileged or relatively affluent, as well as foreigners, especially from China [59]. Globally, several incidents of harassment, physical violence and stigmatization towards healthcare workers have emerged during the course of the Covid-19 pandemic [31]. In our study, there were no incidents of harassment, physical violence and stigmatization reported towards healthcare workers. Similarly, in the weeks preceding the lockdown in Italy, the sentiment toward the Chinese community changed to the extent that it left their restaurants empty, more and more parents did not want their children to go to school if they had a Chinese classmate, and a high-profile politician disparagingly saying on television that “we have all seen them eat live mice” (p.39) [28].

Stigmatizing responses to Covid-19 is often informed by myths and misconceptions. Even though SARS*-*CoV*-*2 is a novel virus, which means that everyone is at risk of contracting the disease as no one has immunity, claims about natural immunity persists [13]. We found that there is a denial of the existence of the disease and that young people believe themselves to be immune to catching Covid-19. Such misconceptions might lead to young people not adhering to preventive measures. They might also not adhere to social distancing measures and ignore restrictions put in place, such as prohibiting gatherings of more than 50 people.

Another myth that emerged from the data is that of “eating the wrong things”.

Currently there is no evidence that food is associated with transmission of the virus [61]. With maize meal considered a staple food in South Africa [62], study findings revealed that key informants perceive eating maize meal porridge, as a protective factor against Covid-19. Drinking hot water was also mentioned by key informants as a cure for Covid-19. Scientific consensus is that patients with coronavirus must have plenty of water as that will keep mucous membranes moist which can further lower the chances of cold and flu [61]. Similarly, according to Arshad and colleagues (2020), drinking plenty of water, taking minerals like magnesium, and zinc, micronutrients, herbs, and food rich in vitamins C, D and E and maintaining a healthier lifestyle can help to overcome the infection [61]. In this way, “eating the wrong things” (and drinking plenty of water) is premised on scientific fact (i.e. a healthy diet might help in the event of exposure to disease) combined with false information.

In our study, eating maize meal porridge was seen by study participants as a protective factor against Covid-19 (i.e. strengthening immune systems) and drinking hot water was perceived as a cure for the disease. Myths and misconceptions regarding a cure for the disease could affect non-adherence to preventive measures because people might not self-isolate/quarantine or test for Covid-19 if they present with any symptoms. Similar study findings were reported in other countries. For instance, in Nigeria, study investigators found that there is a myth that the virus can be killed by drinking alcohol and eating good food to strengthen the immune system [60]. If widespread myths regarding immunity against the virus persists non-pharmaceutical preventive measures such as social distancing in combination with other preventive behaviours including the wearing of masks and frequent hand washing might not be adhered to. Non-adherence to preventive measures can lead to increased surges in Covid-19 infections.

False information observed in the data is that Covid-19 test kits, according to study participants are contaminated with the virus. News24, a South African online news service, reported on 7 April 2020 that a 55-year-old man posted a video on Facebook claiming that Covid-19 testing kits were contaminated, and he urged South Africans to refuse testing for Covid-19 [63]. The man who resides in Cape Town was later arrested by police under the Covid-19 Disaster Management Regulation 11(5), which prohibits and criminalizes the spread of fake news pertaining to efforts of combating this pandemic [63]. This could result in people refusing to test, even though they presented with symptoms of Covid-19. Low uptake of testing for Covid-19 makes it difficult to identify and track positive cases [33].

What this study shows us, is that myths, misconceptions, othering and stigmatizing responses to Covid-19 in South Africa can hamper efforts to mitigate the transmission of the disease. Whatever the source of such myths and misconceptions, it might impact public health efforts to test, track and trace those infected with Covid-19. Although not unique to South Africa, the propagation of myths and misconceptions may also increase resistance to the uptake of a future Covid-19 vaccine, which has already been receiving local pushback based on information generated outside the country.

## Study limitations

The results of this study should be considered in terms of its methodological limitations. Due to the exploratory descriptive qualitative research design, only very tentative generalizations of the study findings are possible. The exploratory descriptive qualitative study design did not allow for any analysis (and comparison) between provinces. Conducting an analysis between provinces would have been misleading due to the small sample size of the study. Purposive sampling was limited to the three high-burden Covid-19 provinces at the time of the rapid assessment. Key informants were mainly recruited through civil society organizations and NGOs. Hence, the method of sampling led to the selective inclusion of individuals who have established networks and ties with the HSRC.

The results apply only to study participants who participated in the assessment. Overall, researchers were able to reflect on some similarities experienced by study participants because of commonalities based on language, race and culture making them an ‘insider’ to some extent, however, all researchers completed a post-graduate education, which placed them as middle class, reinforcing an ‘outsider’ status, to many of our participants’ working class situation.

The study also relied on self-reported information consequently there might be under-and over reporting on sensitive topics. There might be significant over-reporting in the extent to which participants take preventive measures to curb the spread of Covid-19. Due to social desirability bias, participants might over-report that they take precautions at home and in the workplace or social distance to prevent the spread of Covid-19. In addition, to social desirability bias as a potential limitation to interpreting the study findings, there was a risk that study participants might have felt uncomfortable responding to some of the questions posed.

In person or face to face data collection was no longer possible with the onset of the Covid-19 pandemic. Hence, in our study we made use of telephonic interviews. Even though telephonic interviews are common qualitative data collection methods, limitations with regards to making use of this remote data collection method were also apparent. We asked study participants to position themselves in a private space where disruptions of any kind were minimized and also where study participants felt most comfortable in to take part in a telephonic interview without fear of any aspects of their personal lives being revealed to others in the household if they wish to keep it private. There was a risk however that someone outside the study will overhear the interview. We also asked participants to ensure that there are little to no disturbances during the interview because, the interview will be recorded. These requests might have caused inconvenience on the part of the study participants. For instance, study participants might have found it challenging to position themselves in spaces where they can remain undisturbed by other members of the household throughout the telephonic interview, due to household crowding. Finally, qualitative interviews might have been interrupted because of geographical areas where mobile cell phone connectivity was poor.

Despite these limitations, this study brought attention for the first time to the emergence of stigmatizing and othering responses to Covid-19 in South Africa. It is our hope that this exploratory study, will assist in understanding how myths, misconceptions and false information about vulnerability to Covid-19 could potentially affect non-adherence to containment measures that were put into place to curb the transmission of the disease in South Africa.

## Conclusions

The South African government’s swift and scientific response to the management of Covid-19 restored confidence and trust in its ability to manage not only pandemics but also social distress brought on by disease outbreaks. Building from the trust brought on by the government’s swift and scientific response to Covid-19, a broader public dissemination should be conducted through the development of culturally and linguistically tailored communication materials for a range of audiences. Widespread myths and misconceptions should be taken into consideration by government implementers as they design preventive messages. HSRC researchers, government implementers, civil society representatives and community leaders should collaborate in the translation of the study findings, into accessible formats for various target audiences.

Importantly, in the development of communication materials and messaging to the public during disease outbreak, affected and vulnerable groups and communities should be included in the dissemination and development of simplified and targeted messages. Our study findings revealed that younger people do not see themselves to be at risk of catching SARS*-*CoV*-*2. Communication materials specifically tailored to younger people, should incorporate the use of media and social networking sites and platforms. The approach to tailoring communication materials for younger people should allow engagement and mobilization of local influencers in the community sector to address misconceptions and stigmatizing responses to Covid-19. Crucial to the development of media content for younger people to address myths and misconceptions about Covid-19 is the incorporation of socio-cultural experiences of the South African youth in dealing with the Covid-19 pandemic.

Stigmatizing responses to Covid-19 revealed that specific groups (i.e. ‘black’ South Africans) perceive themselves to be immune to catching the virus, whilst blaming other groups for being responsible (and more vulnerable) for the transmission of the disease (i.e. ‘white’, wealthy South Africans and people of Asian descent). Developing an online collaborative platform between scientists and the South African public, to debunk myths and misconceptions about Covid-19 is integral to maintaining preventive measures and correct knowledge about who is most vulnerable to Covid-19. Such an online collaborative platform between scientists and the South African public, should be expanded across Africa for engagement that makes use of language that is inclusive, emphasizing that viruses do not target specific groups of people or ethnicities. Terminology guidelines that makes considerate use of language should be developed with scientists, civil society representatives and representatives of specific groups of people or ethnicities in order to decrease the possibility of stigmatizing specific groups of people. Finally, communication content should be developed to further inform the public health response to Covid-19 using HSRC data to ensure it is accessible to policy makers and wider communities of national and local policy actors, in government and civil society.

## Supporting information

S1 File (DOCX)
