# Supplementary material for: Myths, misconceptions, othering and stigmatizing responses to Covid-19 in South Africa: A rapid qualitative assessment
Source: PLoS One. 2020 Dec 22;15(12):e0244420. doi: 10.1371/journal.pone.0244420 (PMC7755184; doi:10.1371/journal.pone.0244420)
Supplement: S1 File — (DOCX) [file pone.0244420.s001.docx]

# Supporting Information 1. Semi-structured interview guide

**Themes and Probes**

This is comprised of a set of core questions with probes and specific questions for each target group.

1. **General Question (for all participants)**
   1. People are talking about the coronavirus. What do you know or what you have you heard about the coronavirus?

Probe: When did you first hear about the coronavirus? Source of information, do you remember where you heard of the coronavirus first?, what did the information say? Probe for if they heard first about the coronavirus from friends, family, or social media, how did they talk about the virus, other words/terms that are used?)

- 1. Some people are taking the outbreak seriously, others are not?
     1. Probe on when and if they are taking the outbreak of the coronavirus seriously? Why have you started taking it seriously
     2. Probe on what were your first experiences/precautions that you started practicing at your workplace? Traveling to work in a taxi/bus? At home?)
     3. Probe on when social distancing was initiated and with whom? How did it affect your social life?
  2. In your social network, whom are you most concerned of in possibly getting infected? And why?
     1. Outside of your family and friends, are there other people who you think will most likely become infected with the coronavirus? (Probe for why?)
  3. Do you have any experiences with the coronavirus? If yes, tell me more. i.e. do you know anyone who has been infected (got it) in your family, friends or community? If so, who? How close of a family member/friend are they to you?
     1. Have you been in contact with this person since learning of their infection? (Have you seen that person physically and how close were you to that person).

Probe: myths, misconception about the spread and origins too etc.

1. **Prevention (for all participants)**
   1. Do you think it is important for you to be protected from the coronavirus? Tell me more.
   2. What are you doing to protect yourself from being infected with the coronavirus?

Probes: changing behavior, adopting preventative practices, knowledge of prevention, lockdown procedures etc.

2.3 What are people around you doing to protect themselves from being infected with the coronavirus? Do you think they are doing the right thing?

Probe: community initiatives, panic buying, self-isolation, local responses from the government, NGO etc.

2.4 There are different responses/reactions that people have with regards to the lockdown. Probe for impact on family/friends? Positive or negative responses

2.5 Are you employed? Yes/No. if employed, What work do you do? How has the coronavirus affected you regarding your work? Has your employer communicated anything to you about how the coronavirus/how it will affect your work?

2.5.1 Have there been changes to how things are done in your place of work? Tell me about these (e.g. more handwashing, cleaning of surfaces, working from home etc.

2.5.2 Do you believe that this is enough? If yes, why or if No, why? If No, what more would you like to be done?

2.5.3 Probe: options to work from home? Still going to work? Work terminated?

2.6 What worries you the most about the coronavirus?
Probe: Vulnerability, structural issues like housing, close proximity, government response, access to testing, access to care, work, and mode of transport used to get to work or commute, ablution facilities etc.

2.7 If you are a member of a religious organization – how is you religious institution responding to the coronavirus? What are they doing saying about the coronavirus

2.8 Do you believe that this is enough? If yes, why or if No, why? If No, what more would you like to be done?

2.9 What do you think they could do better?

1. **Coping/Managing infections (for all participants)**
   1. If you think you have the symptoms of the coronavirus, do you know what to do?
      1. Could you tell me more and please explain? How did you know what to do?
   2. Statement: Authorities talk about social distancing, self-isolation and tracing of contacts when one is infected.
      1. What have you heard about protecting yourself or anyone who has the coronavirus?

Probe: if they do not mention anything of social-distancing etc., ask

3.2.2 What would you do to help your family if someone (living with you) was infected with the coronavirus?

3.2.3 What would you do to help your community if someone was infected with the coronavirus?

- 1. How are people with coronavirus or those thought to have coronavirus treated in your community?

Probe: stigma and discrimination, fear of reporting etc.

- 1. Do you think that the public is being given all the information about the coronavirus? (probe or is information being kept from us?)
     1. If you think information is being withheld Who do you think is withholding information from us and why?
     2. Do you believe all the information that we are being given?
  2. In you view do you think the government is doing enough to combat the spread of the infection

Probe: access to information, access to water, masks and all related prevention measures.

1. **Immigrant communities**

4.1 Do you think the South African government (at all levels) has made provision to protect you and members of the immigrant community? Please tell me more

4.2 Do you feel that if you suspect that you have the coronavirus, would you be able to access a coronavirus test and be treated? Would you be treated differently to South African citizens (people who were born here)?If so, in what way and how/why?

4.3 Are you aware of anyone who has tested positive in your community? If so, how are those people being treated in your community?

4.4 Which other services would you say is important to you, that you can’t access? (Are you able to access all the services that will assist during this time?)

4.5 How are people who you think may be positive with coronavirus being treated by other in your community? Probe: Are there any information about the corona virus which you think may be doubtful? What myths, misconceptions about the origins and the spread of the corona virus and Covid-19 have you heard? Please could you share them with me?

4.6 What have you heard about the situation relating to the corona virus in your country of birth? How is it being handled? What is the impact? How do you compare the handling of the pandemic there, when compared to the situation in South Africa at this time?

4.7 Are you able to continue with your work or means to make a living at this time? How are you making a living?

4.8 What has been the most challenging situations for you during this time; (coronavirus and the lockdown)?

4.9 How long will you think the lockdown will last? Will the lockdown period be extended? Are people in your community adhering to the lockdown regulations?

4.10 What kind of assistance would you think you will be able to get from the government after the corona virus lockdown is over?

1. **Religious leader**

5.1 How has the coronavirus affected congregation meetings and planned gatherings?

5.2 Are there challenges that the coronavirus has created for you? Tell me more.

5.3 How do you feel about coronavirus hitting humanity? Tell me more

5.4 Does your religious institution have a solution that they think could combat the spread of the virus?

Probe: conventional and spiritual solutions

5.5 Do you feel that if you suspect that you have the coronavirus, would you be able to access a coronavirus test and be treated? Would you be treated differently to those who were not infected?If so, in what way and how/why?

5.6 How are people who you think may be positive with coronavirus being treated in your religious institution?

Probe: myths, misconception about the spread and origins too etc**.**

**6. Traditional leaders:**

6.1 How has the coronavirus affected your daily functioning/gatherings in your community?

6.2 What are the people in the community you serve saying about the coronavirus? Tell me more

6.3 Have you addressed your community regarding the coronavirus? If yes, what did you say to the people? How did you do it? When was it done (rough estimate of date)? Tell me more.

6.4 Do you feel that if you suspect that you have the coronavirus, would you be able to access a coronavirus test and be treated? Would you be treated differently to those who were not infected? If so, in what way and how/why?

1. **Taxi/Buses Owners/Drivers:**
   1. In what ways has the coronavirus impacted your business? Tell me also about how measures being implemented by authorities are affecting your business. Describe to me the ease or difficulty with which you have found implementing the measures.
   2. What measures have you taken to protect yourself and passengers from getting coronavirus? What challenges, if at all, have you faced in taking these protective measures? Tell me more, what do you do Probe: Do you have hand sanitizer for you to use? What about for your passengers?. Do you take precautions and safety such as wipe the vehicle seats and dashboards regularly since the coronavirus outbreak? What about opening windows?
   3. What are your thoughts regarding people coughing/sneezing in taxi/bus? How do you or would you react? Tell me more

Probe: How do you respond to the person

7.4 Do you feel that if you suspect that you have the coronavirus, would you be able to access a coronavirus test and be treated? Would you be treated differently to those who were not infected? If so, in what way and how/why?

**7.1b Taxi commuters**

7.1.1.b Please tell us about your experiences with travelling in taxi’s, since the start of the Coronavirus epidemic?

Probe: regarding whether taxi drivers provide adequate protection, any fears or concerns when getting inside the taxi. What measures do they take or have they been taking personally to protect themselves. What challenges, if at all, have they faced in taking these protective measures?

Probe also regarding how measures being implemented by authorities are seen or experienced, including their adequacy and feasibility / ease of adoption, and whether and how they have posed challenges. During the 2 weeks before lock down, what measures did you take to protect yourself from getting coronavirus when traveling with a taxi? Tell me more.

7.1.2 Do you feel that if you suspect that you have the coronavirus, would you be able to access a coronavirus test and be treated? Would you be treated differently to those who were not infected? If so, in what way and how/why?

**8. Youth (out of school)**

8.1 Being considered a young person what are your thoughts regarding Coronavirus?

8.2 How would you say coronavirus affected your life? If it has at all? Why and why not?

8.2.1 Probe: which online platforms are you getting your information from, WHO, Twitter, Facebook?

8. 3 What/how do you consider facts and fake news regarding the coronavirus?

8. 4Which platforms would you say have fake/true information regarding coronavirus, how can you tell?

8.5 Are you currently doing any further studies? If yes, has the current situation impacted your studies in any way? Access to resources/study material? Accessing study groups

8.5.1 Probe: Any assistance from your place of schooling?

8.6 Has this current situation affected how you go about your friendships/relationships/kinships?

8.7 What has been the most challenging during this time for you and other young people (coronavirus and the lockdown) in your opinion ?

8.8 How do your friends/peers/work colleagues feel about this coronavirus?

8.9. Do you feel that if people suspect that you have the coronavirus that you would you be able to access a coronavirus test and be treated? Would you be treated differently? If so, in what way and how/why?

8.10. If you could advise, what kind of assistance do you think would be better suited for you during this lockdown period?

**9. Vulnerable groups (sex workers, LGBTI+ people, street communities, people living with disabilities)**

9.1 Do you think the government has made provision to protect you in this outbreak of the coronavirus? Tell me more

9.2 Do you feel that if you suspect that you have the coronavirus, would you be able to access a coronavirus test and be treated? Would you be treated differently to those who were not infected? If so, in what way and how/why?

9.3 Which kind of services would you say is important to you that you can’t access? (Are you able to access services that will assist during this time?)

9.4 Are you aware of anyone who has tested positive in your community? If so, how are those people being treated in your community?

9.5 Probe: Are there any myths, misconception about the spread and origins too that you might have heard? Could you share them?**.**

9.6 How are you making a living during this time?

9.7 What has been the most challenging during this time for you personally (coronavirus and the lockdown) in your opinion?

9.8 If you could advise, what kind of assistance do you think would be better suited for you during this lockdown period?

**10. Caregivers of elderly people in old age homes**

10.1 Can you describe your role?

10.2 What are your concerns about safety regarding the COVID-19 pandemic in the workplace for yourself, colleagues and other staff in the old age home?

Probe: training, that elderly are more susceptible to COVID19, resources/facility readiness, availability of sanitizers

10.3 Are you concerned about passing the infection to your family members?  What are you doing to prevent this from happening?

10.4 Are you adequately prepared to interact with the elderly who might have the virus or those who have/are critically ill as a result of the virus? Did you receive specific training for this?

10.5 What are the prevention measures are in place for you, patients and patient’s visitors?  Are these adequate, and feasible and are they being followed?

10.6 Do you think the old age home has made provision to protect you? What about government? What more could be done and by whom?

10.7 Do you feel that if you suspect that you have the coronavirus, would you be able to access a coronavirus test and be treated? Would you be treated differently to those who were not infected? If so, in what way and how/why?

1. **Airport workers**

11.1 Can you describe your role?

11.2 What are your concerns about safety regarding the COVID-19 pandemic in the workplace for yourself, colleagues and other staff at the airport?

Probe: training given or required, readiness of the airport, availability of sanitizers

11.3 Were you adequately prepared to interact with travelers? Please explain

            Do you believe that you are currently adequately prepared to interact with travelers?

11.4 Are you concerned about passing the infection to your family members?  What are you doing to prevent this from happening?

11.5 Do you feel that if you suspect that you have the coronavirus, would you be able to access a coronavirus test and be treated? Would you be treated differently to those who were not infected? If so, in what way and how/why?

1. **Community health care workers**

12. 1 Please describe your role as a community health care worker.

- Probe: how has the coronavirus changed the role you play in your community? In terms of procedures and practices?

12.2 Since the announcement of COVID 19 in South Africa, have you received any form of training about coronavirus?

- Probing: How helpful was the information received from the training?
- how many trainings were offered, where did you receive the training? Have you been educating the community about the virus?

12. 3 What measures have you undertaken to protect yourself as you perform your responsibilities in the community?

- Probe: What are your fears?

12.4 What challenges do you experience in the community as a healthcare worker due to coronavirus?

- Probe: What are your thoughts on the community’s reaction to the coronavirus? Are they observing recommendations for social distancing, washing of hands etc?

12. 5 What support system has been made available for you in performing your responsibilities?

- Probe: support from the clinic etc.?

12. 6 What have been the lessons learnt from the coronavirus concerning your role as a community health care worker?

12.7 Do you feel that if you suspect that you have the coronavirus, would you be able to access a coronavirus test and be treated? Would you be treated differently to those who were not infected? If so, in what way and how/why?

**13. *Shebeen* owners**

13.1 In what ways has the coronavirus impacted your business? Tell me also about how measures being implemented by authorities are affecting your business. Describe to me the ease or difficulty with which you have found implementing the measures.

13. 2 What measures have you taken to protect yourself and clients from getting coronavirus? What challenges, if at all, have you faced in taking these protective measures? Probe for hand sanitizer for you to use? Steps taken around handling of food and surfaces? What about opening windows?

13. 3 What are your thoughts regarding people coughing/sneezing in your shebeen? How do you or would you react? Probe: How do you respond to the person

13.4 Do you feel that if you suspect that you have the coronavirus, would you be able to access a coronavirus test and be treated? Would you be treated differently to those who were not infected? If so, in what way and how/why?

**14. *Shebeen* clients**

14.1 In what ways has the coronavirus impacted you as a *shebeen* client?

14.2 Please tell us about your experiences with regard to using using *shebeens* since the start of the Coronavirus epidemic? Probe: regarding whether owners provide adequate protection, any fears or concerns when visiting a *shebeen.* What measures do they take or have they been taking personally to protect themselves. What challenges, if at all, have they faced in taking these protective measures?

14.3 What are your thoughts regarding people coughing/sneezing in the *shebeen*? How do you or would you react? Probe: How do you respond to the person

14. 4 Do you feel that if you suspect that you have the coronavirus, would you be able to access a coronavirus test and be treated? Would you be treated differently to those who were not infected? If so, in what way and how/why?

**15. Teachers questions**

15.1 How did your school react/respond to the coronavirus outbreak?

15.2 Are there any measures that were taken for prevention or awareness? For the learners or staff.

15.3 Did your school have any learners/teachers that may have been exposed to the corona virus from recent travels?

15.4 Did you have learners/teachers who have tested positive for the corona virus?

15.5 Did you have learners whose parents or family members tested positive for the virus?

15.6 How has the coronavirus outbreak affected your planned teaching schedule for the current year?

15.7 Has your school made any provisions to assist you with the interrupted schedule?

15.8 What are some of the systems that have been put in place to mitigate for the loss of teaching time due to the early closure of schools and the national lockdown

15.9 Was there any training given on how to assist learners/teachers in how to use the new systems and technologies?

15.10 Do you feel that if you suspect that you have the coronavirus, would you be able to access a coronavirus test and be treated? Would you be treated differently to those who were not infected? If so, in what way and how/why?

**16. Pregnant Women**

16.1 How has the coronavirus impacted your pregnancy if at all?

16.2 With the onset of the coronavirus has your doctor/nursing staff advised you on how you can better protect yourself during your pregnancy?

16.3 Has your hospital/clinic made any provisions to assist you during your pregnancy?

16.4 What has been the most challenging situations for you during this time; (coronavirus and the lockdown)?

16. 5 What are some of your fears, concerns during this outbreak and of being pregnant?

For information about the Corona virus please visit: <https://www.who.int/emergencies/diseases/novel-coronavirus-2019/advice-for-public>

We can send this website link to you on WhatsApp at the end of this interview.

For further information on the Corona virus from the Department of Health you can set up the Covid-19 Connect, which is a WhatsApp service, on your phone

Step 1: Save the number 0600 123456 to your contacts on your cellphone.

Step 2: In WhatsApp send the word "Hi" to Covid-19 Connect and start chatting.

Alternatively, you can call the department's coronavirus hotline on 0800 029 999.
